# Short-Term Environmental Enrichment Enhances Adult Neurogenesis, Vascular Network and Dendritic Complexity in the Hippocampus of Type 1 Diabetic Mice

**DOI:** 10.1371/journal.pone.0013993

**Published:** 2010-11-15

**Authors:** Juan Beauquis, Paulina Roig, Alejandro F. De Nicola, Flavia Saravia

**Affiliations:** 1 Institute of Biology and Experimental Medicine, National Research Council (CONICET), Buenos Aires, Argentina; 2 Department of Biochemistry, Faculty of Medicine, University of Buenos Aires, Buenos Aires, Argentina; Centre de Recherches sur la Cognition Animale - Centre National de la Recherche Scientifique and Université Paul Sabatier, France

## Abstract

**Background:**

Several brain disturbances have been described in association to type 1 diabetes in humans. In animal models, hippocampal pathological changes were reported together with cognitive deficits. The exposure to a variety of environmental stimuli during a certain period of time is able to prevent brain alterations and to improve learning and memory in conditions like stress, aging and neurodegenerative processes.

**Methodology/Principal Findings:**

We explored the modulation of hippocampal alterations in streptozotocin-induced type 1 diabetic mice by environmental enrichment. In diabetic mice housed in standard conditions we found a reduction of adult neurogenesis in the dentate gyrus, decreased dendritic complexity in CA1 neurons and a smaller vascular fractional area in the dentate gyrus, compared with control animals in the same housing condition. A short exposure -10 days- to an enriched environment was able to enhance proliferation, survival and dendritic arborization of newborn neurons, to recover dendritic tree length and spine density of pyramidal CA1 neurons and to increase the vascular network of the dentate gyrus in diabetic animals.

**Conclusions/Significance:**

The environmental complexity seems to constitute a strong stimulator competent to rescue the diabetic brain from neurodegenerative progression.

## Introduction

In both humans and animal models, diabetes mellitus is associated with pathological changes in the central nervous system that lead to cognitive and affective deficits and to an increased risk for brain vascular complications [1], [2]. In animal models of diabetes, several brain alterations have been described, such as increased hippocampal astrocytic reactivity, impaired synaptic plasticity, vascular changes, decreased dendritic complexity and disturbed neurotransmission [3]–[7]. Also, alterations in hippocampal-dependent learning tasks were found in diabetic rodents [6], [8]. Reductions in adult hippocampal neurogenesis, defined as the process of generating and integrating new neurons in specific and restricted areas of the brain including the dentate gyrus, were described in different models of diabetes [9]–[11], as well as in aging, stress and inflammation [12]–[14]. The decline of hippocampal adult neurogenesis has been associated with impaired hippocampal dependent learning [15], [16] Granule neurons, glia and blood vessels located at the subgranular zone of the dentate gyrus are components of a microenvironment often called ‘neurogenic niche’ where progenitors reside and new neurons are generated [17], [18] and an alteration in any of these elements can affect neurogenesis. Decreased cerebral blood flow and loss of vasodilatory stimuli are vascular disturbances commonly found in the diabetic brain [19], [20], suggesting possible disturbances to the neurogenic niche. We have previously shown that the reduction of adult neurogenesis in diabetic mice can be prevented by short term hormonal and antidepressant treatments [21], [22].

Reductions of hippocampal neuronal dendritic length and complexity were found in association with aging and stress [23], [24]. Other authors have reported a reduction in length and a simplification of dendritic trees of hippocampal pyramidal cells in diabetic rodents [4], [25]. There is evidence from animal models showing that changes in dendritic morphology, probably associated with synaptic disturbances, correlate with alterations in memory and learning abilities [26], [27].

The positive influence of environmental enrichment on learning and memory in laboratory animals has been known for more than 60 years [28], [29]. There are studies showing that adult hippocampal neurogenesis, dendritic complexity of hippocampal neurons and some learning abilities can be improved by housing animals in an enriched environment [30]–[34].

In the present work, we present results obtained using the streptozotocin-induced diabetes model, a widely used model of irreversible type 1 diabetes. Streptozotocin is an alkylating agent that damages DNA and causes cell death. The drug is transported into the cell via the GLUT2 glucose transporter, damaging pancreatic beta cells in a highly selective manner due to the high levels of this type of glucose transporter in this cell population [35].

Using histochemical and immunohistochemical techniques, bromodeoxyuridine incorporation, a modified version of the Golgi silver impregnation technique [36] and Sholl analysis [37] we investigated hippocampal neurogenesis, the vascular network of the dentate gyrus and the dendritic complexity of CA1 pyramidal cells in streptozotocin-induced type 1 diabetic mice. We studied the effects of an environmental enrichment protocol on diabetes-associated brain changes. Our results showed an effect of the environmental stimulation on proliferation, differentiation and survival of newborn granular neurons in the dentate gyrus from diabetic mice. The CA1 pyramidal neurons also exhibited increased dendritic arborization and spine density. Accompanying these phenomena, an enhancement of the vascular network area of the dentate gyrus was found.

## Materials and Methods

### Animals

Adult male C57BL/6 mice of 16 weeks of age (27–30 g) were obtained from the Institute of Biology and Experimental Medicine Animal Facility (NIH Assurance Certificate # A5072-01) and were housed under controlled conditions of temperature (22°C) and humidity (50%) with 12 h/12 h light/dark cycles (lights on at 7:00 am). All animal experiments followed the NIH Guide for the Care and Use of Laboratory Animals and were approved by the Ethical Committee of the Institute of Biology and Experimental Medicine. This Ethical Committee is composed by Ricardo Calandra, MD, PhD (Head); Alberto Baldi, MD, PhD; Carlos Libertun, MD, PhD; Enrique Segura MD, PhD and Claudia Marro Psychologist, all members of the National Research Council (Argentina). All efforts were made to minimize animal suffering and to reduce the number of mice used.

### Streptozotocin administration and differential caging conditions

**Figure 1:**
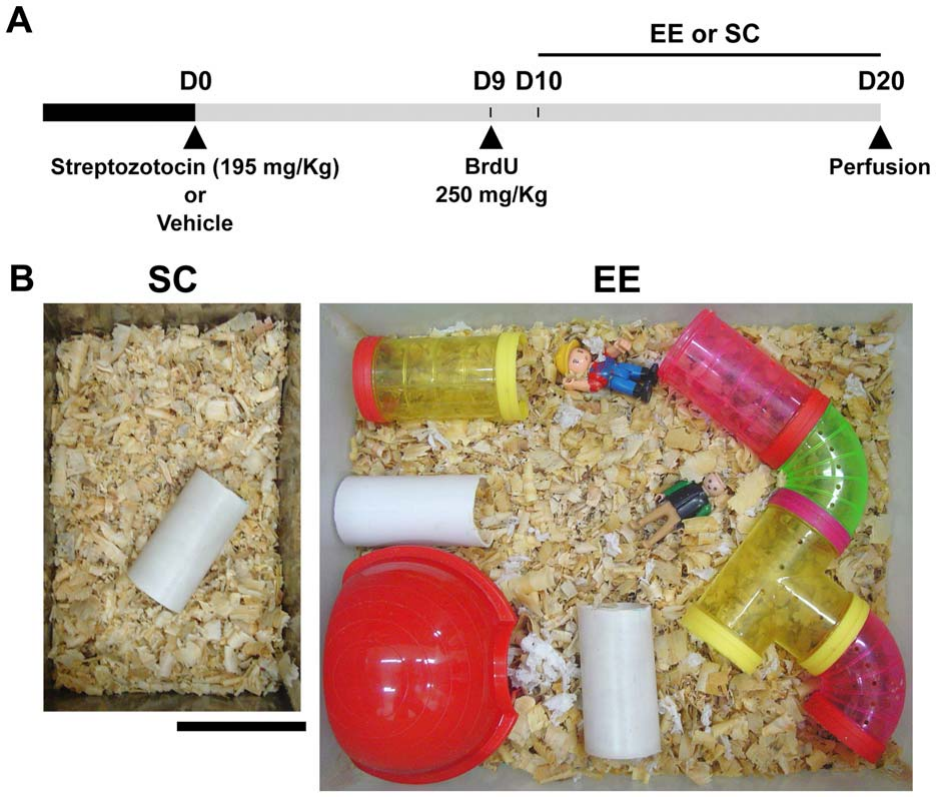
Experimental protocol and caging conditions. (A) At 16 weeks of age, mice were injected with streptozotocin or vehicle correspondingly (day 0). At day 9, bromodeoxiuridine was administered i.p in order to label dividing cells. At day 10, mice were differentially housed under standard (SC) or enriched (EE) conditions until day 20 when perfusion was done. (B) Experimental cages. In the left panel a photograph of the standard caging (SC) condition is shown. A single plastic tube was included to reduce aggression between mice. The right panel corresponds to the environmental enrichment (EE) condition. Tunnel, toys, plastic houses and nesting material were provided. Scale bar corresponds to 10 cm.

Mice were injected intraperitoneally with streptozotocin (Sigma, 195 mg/kg) or vehicle (citrate buffer 0.1 M, pH 4.5) and were tested for glycosuria 48 h later using Keto-Diastix (Bayer Diagnostics, Argentina). Following a positive urine test, mice were assigned to the diabetic groups (n = 10) or, those with negative urine test that were injected with vehicle, to control groups (n = 10). Ten days after the streptozotocin injection, half of the animals in each group were separated into standard caging and enriched environment (Fig. 1-A). In both standard and enriched conditions, water and regular rodent chow were available *ad libitum* and the floor was covered with 2 cm of wooden chips. The dimensions of standard cages (Fig. 1-B) were X = 17.5 cm Y = 27.5 cm, (481.25 cm^2^), Z (height)  = 15 cm with 2–3 mice per cage. In these cages a single plastic tube was placed to reduce anxiety and aggressive behavior [38]. The enriched environment consisted of larger cages measuring X = 40 cm, Y = 33 cm (1320 cm^2^), Z = 15 cm with 5 mice per cage. Toys, extra nesting material, small plastic houses and tubes were available in the enriched condition, with a rearrangement of elements every 2 days. Running wheels were not provided to minimize the influence of physical exercise on neurogenesis. Mice remained in the assigned caging condition for 10 days. Body weight was determined at the day of injection, before starting the differential caging and before perfusion. Glycemia was measured in tail blood using a glucometer (Accutrend GC, Boehringer Mannheim, Germany). Animals with a non-fasting glycemia higher than 14 mM were classified as overtly diabetic.

### Bromodeoxyuridine injection paradigm and tissue processing

To study survival of newborn cells and the effects of caging conditions, we injected bromodeoxyuridine (BrdU, MP Biomedicals) intraperitoneally at 250 mg/kg 24 h before separating mice into the different caging conditions (Fig. 1-A). Ten days after starting the differential caging, animals were anesthetized with a ketamine overdose (333 mg/kg BW, i.p.; Holliday-Scott, Argentina) and then transcardially perfused with 30 ml of 0.9% saline followed by 30 ml of 4% paraformaldehyde in 0.1 M phosphate buffer, pH 7.4. Brains were removed from the skull and dissected. The right hemispheres were assigned to immunohistochemistry procedures and the left hemispheres were processed for the Golgi silver impregnation technique.

### Immunohistochemistry procedures

Right brain hemispheres were fixated overnight in paraformaldehyde at 4°C and then cut coronally at 60 µm in a vibrating microtome. Sections were stored in a cryoprotectant solution (25% glycerol, 25% ethylene glycol, 50% phosphate buffer 0.1 M pH 7.4) at −20°C until use. All immunohistochemical techniques were performed on free-floating sections from 5 mice per group. To study cell survival and neuronal differentiation BrdU immunohistochemistry and double BrdU-β-III-Tubulin immunofluorescence were used. For denaturizing DNA, sections were incubated in a pre-warmed solution of 0.5 M formamide in SSC buffer (0.37 M NaCl, 37 mM sodium citrate) at 65°C for 10 min, incubated in 2 M HCl at 37°C for 30 min and rinsed in 0.1 M borate buffer, pH 8.5, for 10 min. Then sections were blocked for 30 min in TBS with 0.1% Triton X-100 and 10% goat serum and incubated for 48 h at 4°C in a clinical rotator with rat monoclonal anti-BrdU antibody (1∶200; OBT-0030, Accurate Chemicals, Westbury, NY, USA) diluted in blocking solution. Those sections used for double immunofluorescence were co-incubated with monoclonal mouse anti-β-III-tubulin antibody (TuJ1, 1∶1000; G-7121, Promega, Madison, WI, USA). For single BrdU detection, an incubation with a biotinylated anti-rat IgG (1∶200, Sigma), in 0.1% Triton X-100-TBS for 2 hours at RT was performed, followed by processing with the ABC kit (Vector Laboratories, Burlingame, CA, USA) and development with 2 mM diaminobenzidine (Sigma) and 0.5 mM H_2_O_2_ in 0.1 M Tris buffer at RT. For double immunofluorescence, sections were incubated for 2 h at RT with a FITC anti-rat IgG (1∶200, Sigma) and a TRITC anti-mouse IgG (1∶200, Sigma). To study cell proliferation, immunohistochemistry for Ki67 was used. Antigen retrieval was performed by heating sections in 0.01 M citrate buffer, pH 6, for 40 minutes in a thermostatic bath at 85°C. Endogenous peroxidase was inhibited and after blocking sections in TBS with 0.5% Triton X-100 and 10% goat serum, incubation was performed overnight with rabbit polyclonal anti Ki67 antibody (Ki67P Novocastra, UK). For detection we used a biotinylated anti-rabbit IgG (Vector Labs) followed by ABC and diaminobenzidine as described above.

To study dendritic maturation of newborn neurons we used doublecortin (DCX) immunohistochemistry. To block endogenous peroxidase and to enhance the staining of dendritic processes, sections were exposed for 10 minutes to 3 mM H_2_O_2_ in methanol:PBS 50∶50. After blocking unspecific antigenic sites with 10% rabbit serum in PBS, sections were incubated overnight with goat polyclonal anti-DCX antibody (1∶250; sc-8066, Santa Cruz Biotechnology) in PBS with 0.15% Triton X-100 and 1% rabbit serum. For detection we used a biotinylated anti-goat IgG (Sigma) followed by ABC and diaminobenzidine as described above.

### Nissl staining

In order to quantify the total number of granular neurons in the SGZ-GCL, a Nissl staining was performed on brain sections prepared using the same fixation and cutting protocol described for immunohistochemical procedures. Cryoprotected sections were thoroughly rinsed in PBS, put on gelatin-treated slides and air-dried overnight. Then, slides were briefly washed in distilled water and then were immersed in a 0.5% cresyl violet solution for 10 minutes. After that, sections were dehydrated through a series of graded ethanol (70% 2×1 minute each, 95% 2×1 minute each and 100% 2×2 minutes each) and cleared in xylene (2×3 minutes each). Slides were coverslipped using Permount.

### Cell counting

Quantification of the number of BrdU, Ki67 and DCX positive cells was done with a 40x objective in a Zeiss Axioplan microscope. Every eighth 60 µm coronal brain section throughout the entire rostrocaudal extension of the dentate gyrus (DG), corresponding to plates 6–23 and A1100–A3450 from the stereotaxic atlas of the mouse brain [39], were analyzed in each mouse. Cells positive for Ki67 were counted in the subgranular zone (SGZ), defined as a two nucleus-wide band in the limit between the hilus and the granular cell layer (GCL), whereas cells positive for BrdU were counted in the SGZ and in the GCL. Both upper and lower blades of the GCL and SGZ were considered for cell counting. Cells in the uppermost focal plane were not counted. The total number of cells was estimated using the formula T = (N×V)/t, where N is the cell density, V is the volume of the structure (SGZ and GCL) and t the thickness of the section. The volume was calculated by measuring the area of the structure and multiplying by the section thickness and inter-section distance. The area of the SGZ-GCL was calculated on images acquired with a Canon G10 digital camera coupled to a Zeiss Axioplan microscope at 40X. Using NIH software ImageJ [40], photographs were spatially calibrated and profiles of the SGZ-GCL for each section analyzed were manually traced, obtaining the area in µm^2^.

Sections processed for double BrdU-TuJ1 immunofluorescence were analyzed using a Nikon Eclipse E 800 confocal scanning laser microscope with a motorized Z stage. A minimum of 50 BrdU positive cells per mouse were counted in the SGZ and GCL and, as the markers were located in different cell compartments (BrdU in the nucleus and TuJ1 in the cytoplasm), cells were exhaustively analyzed for double staining with tridimensional serial reconstruction. Each cell was studied under a 40x objective followed by a serial reconstruction, using Nikon ezc1 software, based in a series of 25 images along the Z axis with a step of 1 µm each. Cells with a BrdU positive nucleus and a TuJ1 positive surrounding soma were considered as double labeled. Results were expressed as the proportion of BrdU positive cells with TuJ1 co-staining.

### Analysis of the DCX immunohistochemistry

For the quantification of the number of DCX-positive cells two subpopulations were considered following previously published classifications [41], [42]. A less mature population (named A-D) corresponding to cells with not evident or short dendritic processes, with dendrites projecting parallel to the GCL longitudinal axis or presenting a dendritic tree not exiting the GCL. A second, postmitotic, more mature population (named E-F) consisted in cells showing a thick dendrite that reached the molecular layer and a well-developed dendritic tree. For the analysis of the dendritic length of DCX+ cells, at least 25 DCX positive granular neurons in the dentate gyrus were analyzed per brain. Images of the doublecortin positive cells were obtained under a 40X objective using an Olympus BH-2 microscope with a Panasonic GP-KR222 camera. Dendritic processes were traced using Optimas 6.0 image analysis software. Only neurons belonging to the mature subpopulation (E-F) showing a well-developed apical dendritic tree, with a complete and regular staining and not overlapping with neighboring cells were selected for counting. Total length of the immunopositive dendritic tree was measured with Optimas 6.0 analysis software.

### Analysis of Nissl stained sections

The total number of granule cells in the GCL of the hippocampus was estimated using the optical dissector method [43] on Nissl-stained sections. Random sampling was done using counting frames measuring 15 µm ×15 µm ×40 µm (X×Y×Z) at 600× magnification. Cells in the uppermost focal plane and/or intersecting the exclusion boundaries of the counting frame were not counted. The total number of cells was estimated using the formula T = (N × V)/t and the volume of the GCL was measured as explained above.

### Golgi technique and morphological analysis of pyramidal cells

A modified osmium-free version of the Golgi technique was used to study hippocampal pyramidal neurons [36]. After dissection, left hemispheres of mice brains were immersed in 4% paraformaldehyde for 2 hours and then transferred to a solution of 0.95 mM potassium dichromate in 80 mM formaldehyde diluted in distilled water for 48 hours. Then, the tissue was immersed overnight in a solution of 0.95 mM potassium dichromate in 20 mM formaldehyde and then in a solution of 1.2 mM potassium dichromate in distilled water where the tissue remained for 5 days. After that, impregnated tissue was incubated in 0.44 mM silver nitrate in distilled water for 48 hours. All the preceding steps were done in the darkness and at RT. Finally tissue was stored in 30% sucrose in 0.1 M phosphate buffer at 4°C until coronal sectioning with a vibrating microtome at 200 µm [44]. Sections were mounted on slides, pressed with blotting paper to avoid detachment during ethanol dehydration and xylene clearing, and coverslipped with Permount (Fisher Scientific).

At least 15 CA1 pyramidal neurons per animal were traced with a camera lucida attached to an Olympus BH-2 microscope with a 20X objective. Only those neurons showing a completely impregnated dendritic tree and that were relatively isolated from neighboring cells were selected for the analysis. Drawings were scanned with a Hewlett-Packard Scanjet 3610 scanner and then scaled and analyzed with NIH software ImageJ running the Sholl Analysis Plugin v1.0 (written by Tom Maddock and available at http://biology.ucsd.edu/labs/ghosh/software/index.html). This technique [37] consists in superimposing a grid with concentric rings or shells distributed at equal distances ***d*** centered in the soma of a neuron. The number of dendritic intersections ***i*** per shell is computed and branching complexity is evaluated. Dendritic length is estimated by the sum of the products of ***d*** by ***i*** for each ring.

Also the density of dendritic spines was measured in the pyramidal neurons in CA1. Using an Olympus BH-2 microscope with 1250× magnification, dendritic spines were counted in basal and apical, secondary order dendrites. A minimum of 15 pyramidal neurons with dendritic segments at least 20 µm long in the same focal plane were sampled and results were expressed as number of spines per µm.

### Analysis of Lectin-positive blood vessels in the hippocampus

An adaptation of the quantifying method published by van Praag and colleagues was done to determine the lectin positive area in the DG [45]. Images of the DG processed for lectin histochemistry were obtained with a Panasonic GP-KR222 CCD camera on an Olympus BH-2 microscope at 40× magnification and analyzed using Optimas 6.5 software. The contour of the DG was manually traced in 6 sections per animal and the reference area was determined. Using thresholding and automatic area finder tools, the lectin-positive blood vessels were traced and their area measured. The threshold level was determined by an operator blind to the experimental group to allow positive elements to be included and to exclude background signal. It is worth noticing that lectin histochemistry specifically detected vascular elements. The result is expressed as the percentage of the DG area covered by blood vessels.

### Statistics

Data are expressed as the mean ± SEM. Statistical analysis was performed using a two-way ANOVA, followed by Bonferroni's *post hoc* test, with *p*<0.05 as the criterion for statistical significance. Factors considered were ‘glycemic condition’ (control or diabetic mice) and ‘housing’ (standard caging or enriched environment). A repeated-measures (RM) ANOVA with Bonferroni's *post hoc* test was applied for the Sholl analysis. Analysis was done using Prism 3.02 software (GraphPad Software Inc.).

## Results

### Physiological data

Body weight of mice at the beginning of the experiment was not statistically different between groups. At day 20 of the experiment, diabetic groups had a significantly reduced body weight compared with controls: CTL-SC (control mice in standard caging)  = 29.46±1.15, CTL-EE (control mice with environmental enrichment)  = 29.96±0.56, DIAB-SC (diabetic mice in standard caging)  = 24.56±0.42, DIAB-EE (diabetic mice with environmental enrichment)  = 24.36±1.15 (*F*_condition(1,16)_ = 34.82, *p*<0.001; *post hoc* test *p*<0.01 CTL-SC vs. DIAB-SC and *p*<0.001 CTL-EE vs. DIAB-EE); body weight expressed in grams. Type of caging had no significant effect on body weight. Glycemia was evaluated at day 20 and diabetic mice had significantly higher levels than mice in control groups: CTL-SC = 11.24±0.85, CTL-EE = 9.61±0.65, DIAB-SC = 38.71±3.62, DIAB-EE = 28.84±3.09 (*F*_condition(1,16)_ = 91.34, *p*<0.001); glycemia expressed in mg/dL. A ‘housing’ effect was found in diabetic animals (*F*_housing(1,16)_ = 5.539, *p*<0.05; *post hoc* test *p*<0.05 DIAB-SC vs. DIAB-EE). Nevertheless, mean glycemia was above 28 mM in both diabetic groups (SC and EE).

### Cell proliferation, survival and neuronal differentiation

**Figure 2:**
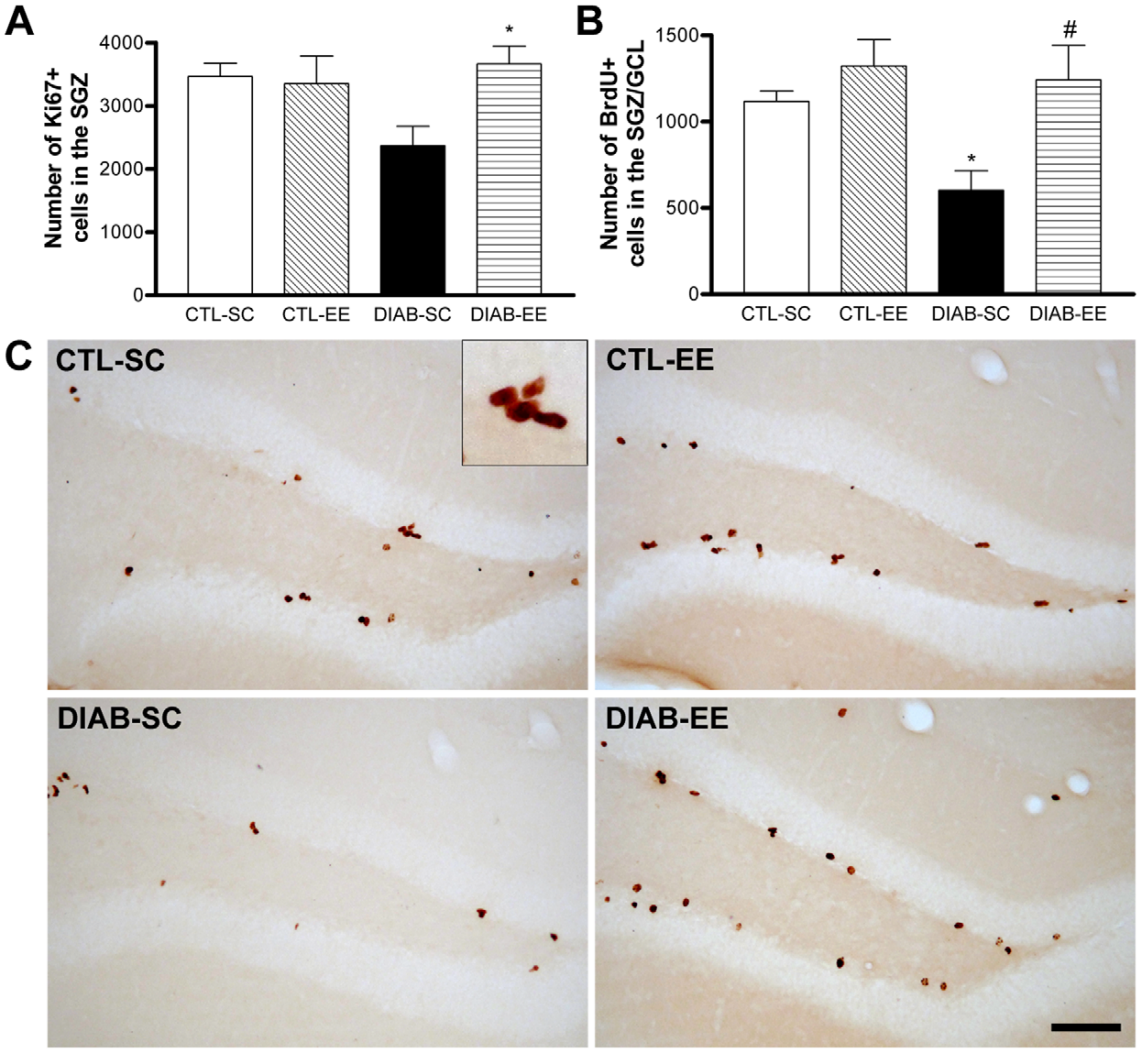
Cell proliferation and survival. (A) Quantification of the number of Ki67-positive cells in the subgranular zone (SGZ) of the dentate gyrus. The DIAB-EE group showed an increase compared with the DIAB-SC group (* *p*<0.05; *post hoc* test *p*>0.05 for every other group pair comparison) (B) Quantification of the number of BrdU-positive cells in the SGZ and granular cell layer (GCL) of the dentate gyrus labeled 10 days after the BrdU injection. The DIAB-SC group showed a decrease in the BrdU-positive cell survival compared with the CTL-SC (* *p*<0.05). An increase was found in the DIAB-EE group (# *p*<0.05 vs. DIAB-SC). (C) Representative microphotographs of the dentate gyrus with anti-BrdU immunohistochemistry. The inset in (A) shows a high magnification view of a cluster of BrdU-positive cells in the SGZ. Scale bar corresponds to 100 µm.

**Figure 3:**
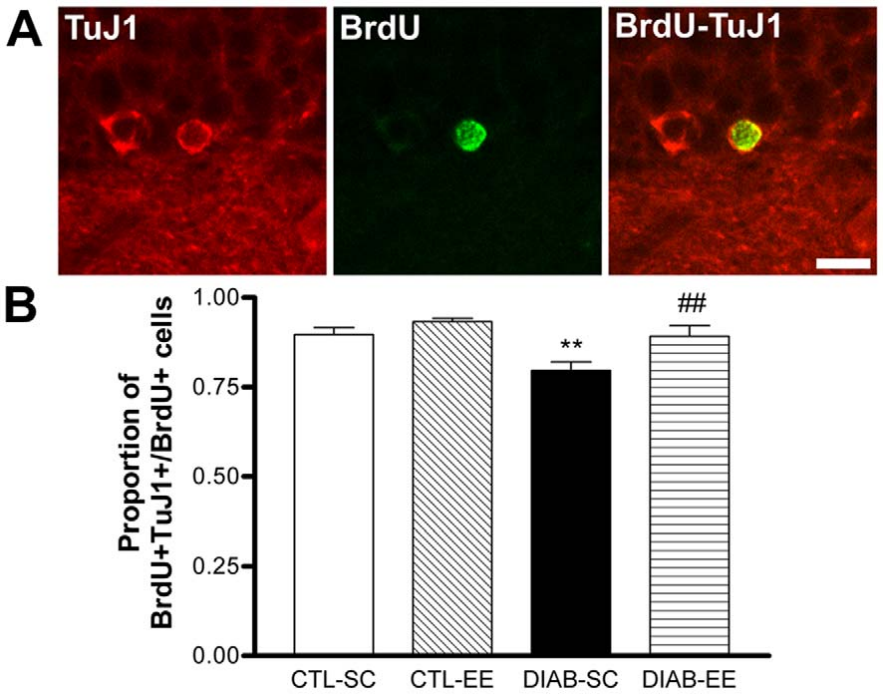
Neuronal differentiation of the BrdU-positive newborn cells. (A) Confocal microscope image of a newborn cell in the subgranular zone (SGZ) showing double labeling for TuJ1 (left panel) and BrdU (center panel). In the right panel a merged image is shown. Scale bar corresponds to 10 µm. (B) Quantification of the percentage of BrdU-positive cells showing co-staining for TuJ1. The percentage was significantly decreased in the DIAB-SC group (** *p*<0.01 vs. CTL-SC) with DIAB-EE mice near control levels (## *p*<0.01 vs. DIAB-SC).

Immunohistochemistry for Ki67 antigen was used to study cell proliferation in the SGZ. An interaction was found between the diabetic condition and the housing (*F*_interaction(1,16)_ = 4.87, *p<*0.05; *F*_condition(1,16)_ = 3.43, *p* = 0.08; *F*_housing(1,16)_ = 1.506, *p* = 0.23; Fig. 2-A). The *post hoc* test revealed a significant difference between DIAB-SC and DIAB-EE groups (*p*<0.05; *post hoc* test *p*>0.05 for every other group pair comparison). Using the BrdU injection/detection technique, cell survival was evaluated in the SGZ and GCL. The cell number was found diminished in diabetic animals in SC compared with controls in the same housing condition (*post hoc* test *p*<0.05 CTL-SC vs. DIAB-SC). This reduction of the cell survival was prevented by the EE (*post hoc* test *p*<0.05 DIAB-SC vs. DIAB-EE; Fig. 2-B and 2-C). No differences in the volume of the SGZ-GCL were found between the experimental groups: CTL-SC 2,895×10^8^±1,471×10^7^, CTL-EE 3,219×10^8^±1,507×10^7^, DIAB-SC 3,051×10^8^±7,155×10^6^, DIAB-EE 3,152×10^8^±1,322×10^7^, expressed in µm^3^. Using double labeling for BrdU and TuJ1, differentiation of newborn cells into a neuronal phenotype was assessed. Neuronal differentiation, determined as the percentage of BrdU-positive cells also stained for TuJ1, was significantly reduced in diabetic animals in SC (*F*_condition(1,16)_ = 11.42, *p*<0.01, *post hoc* test *p*<0.01 CTL-SC vs. DIAB-SC) and environmental enrichment was able to increase the differentiation to control levels in diabetic mice (*F*_housing(1,16)_ = 6.563, *p*<0.05; *post hoc* test *p*<0.01 DIAB-SC vs. DIAB-EE; Fig. 3) finding a significant interaction between factors (*F*_interaction(1,16)_ = 7.213, *p*<0.05).

### Analysis of doublecortin-positive neurons

**Figure 4:**
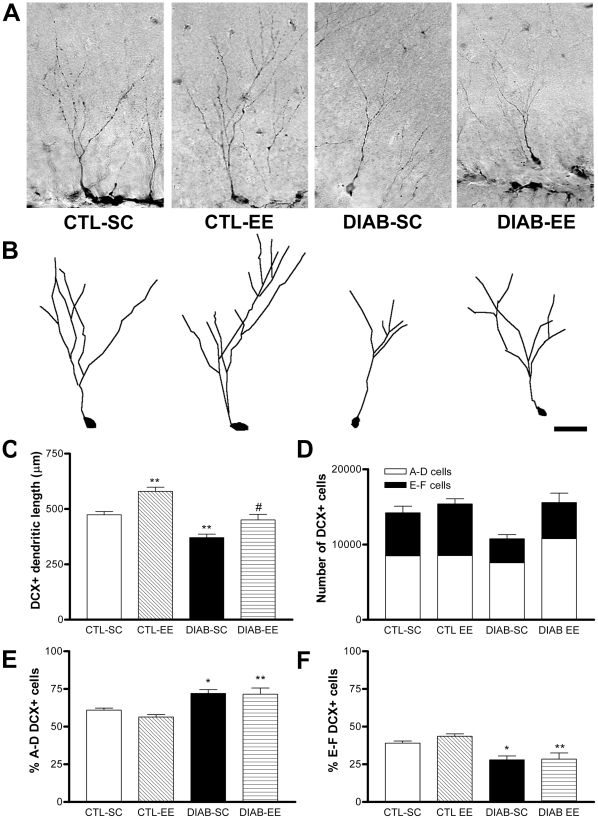
Doublecortin (DCX) immunostaining. (A) Microphotographs corresponding to the DCX immunohistochemistry in the dentate gyrus. (B) Tracing of the dendritic tree of the DCX-positive neurons shown in panel A. Scale bar corresponds to 50 µm. (C) Quantification of the dendritic length of E-F-type DCX-positive neurons. Shorter dendritic trees were found in DIAB-SC mice (** *p*<0.01 vs. CTL-SC) and environmental enrichment prevented this reduction in the diabetic group (# *p*<0.05 DIAB-SC vs. DIAB-EE) and, also, in control mice (** *p*<0.05 CTL-SC vs. CTL-EE). (D) Quantification of the number of DCX-positive cells in the subgranular zone and granular cell layer. No differences were found between groups in the number of total DCX-positive cells and of A-D-type cells. We found a main effect of the ‘glycemic condition’ decreasing the number of E-F-type DCX-positive cells (*p*<0.01). (E) and (F) Percentage of DCX-positive cells showing an A–D (E panel, less mature) and an E–F phenotype (F panel, more mature; see the *Materials and methods* section for further details). Diabetic animals showed an increased percentage of A–D cells and a concomitant reduction in the E–F percentage (* *p*<0.05 DIAB-SC vs. CTL-SC and ** *p*<0.01 DIAB-EE vs. CTL-EE).

Doublecortin-positive cells were classified into two different groups according to maturity as it was already reported [42]. Dendritic length of DCX-positive neurons, assessed in the mature subpopulation (E-F), revealed a significant reduction in diabetic animals in SC compared with controls in the same housing condition (*F*_condition(1,16)_ = 44.46, *p*<0.001; *post hoc* test *p*<0.01 CTL-SC vs. DIAB-SC; Fig. 4). Environmental enrichment increased the dendritic length in both control and diabetic groups (*F*_housing(1,16)_ = 28.06, *p*<0.001; *post hoc* tests *p*<0.05 DIAB-SC vs. DIAB-EE and *p*<0.01 CTL-SC vs. CTL-EE). The total number of DCX-positive cells in the SGZ-GCL from diabetic animals showed a tendency to decrease in mice housed in SC compared with controls in SC and to increase with environmental stimulation (Fig. 4-D) although these differences were not statistically significant. The analysis of each DCX subpopulation revealed a main effect of diabetes decreasing the number of mature (E-F type) cells (*F*_condition(1,16)_ = 6.718, *p*<0.01) with no differences in the *post hoc* test.

The percentage of mature DCX-positive cells (% of total DCX-positive cells) was also decreased in diabetic mice (*F*_condition(1,16)_ = 25.12, *p*<0.001; *post hoc* test *p*<0.05 CTL-SC vs. DIAB-SC and *p*<0.01 CTL-EE vs. DIAB-EE; Fig. 4 E–F). We did not find effects of the environment between both glycemic conditions (*F*_housing(1,16)_ = 0.928).

### Total number of granular cells in the GCL

No statistically significant differences were found in the total number of granule cells in the GCL of the hippocampus between the four experimental groups: CTL-SC 3.723×10^5^±0.24×10^5^, CTL-EE 3.519×10^5^±0.227×10^5^, DIAB-SC 3.164×10^5^±0.108×10^5^, DIAB-EE 3.326×10^5^±0.152×10^5^; total number of granule cells.

### Sholl analysis of CA1 pyramidal neurons

**Figure 5:**
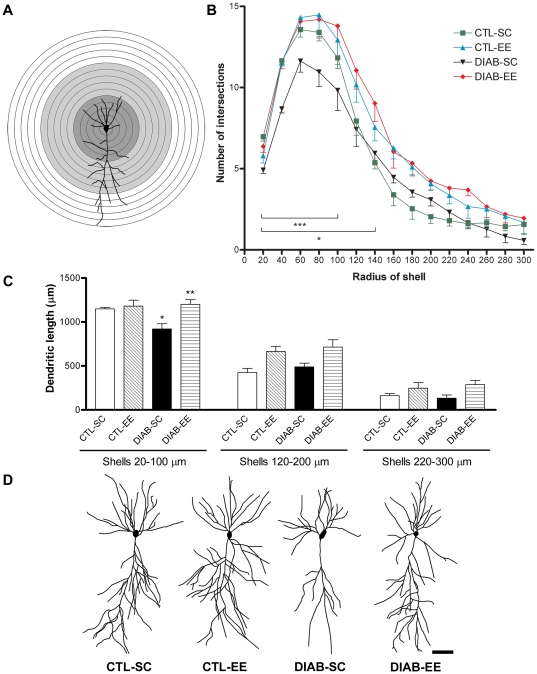
Golgi staining and Sholl analysis of pyramidal neurons in CA1. (A) A camera lucida drawing of a pyramidal neuron and superimposed concentric circles used for Sholl analysis. The radius interval between circles was 20 µm per step, ranging from 20 µm to 300 µm from the center of the neuronal soma. Ranges analyzed for dendritic length: 20–100 µm range (dark grey zone), 120–200 µm range (light grey zone) and 220–300 µm (white zone). (B) Quantification of the number of intersections per circle. Significant differences were found in the circles ranging from 20 µm to 100 µm in DIAB-SC compared with CTL-SC (*** *p*<0.001) and an increase when comparing DIAB-EE with DIAB-SC at 20–140 µm (* *p*<0.05). (C) Quantification of the dendritic length of CA1 pyramidal neurons for the three distance ranges studied: 20–100 µm, 120–200 µm and 220–300 µm. Significant differences were found in the 20–100 µm range with a decrease in DIAB-SC mice (* *p*<0.05 vs. CTL-SC) and an increase in the DIAB-EE group (** *p*<0.01 vs. DIAB-SC). A main effect of ‘housing’ was found at 120–200 µm and 220–300 µm ranges (*p*<0.01 and *p*<0.05 respectively). (D) Camera lucida drawings of pyramidal neurons representative of each experimental group. Scale bar corresponds to 50 µm.

Sholl analysis of CA1 pyramidal neurons stained with the Golgi technique allowed the quantification of branching and length of the dendritic tree (Fig. 5). Diabetic animals had significantly fewer dendritic intersections than controls at the proximal 20–100 µm region (*F*_condition(1,60)_ = 56.6, *p*<0.0001; *post hoc* test *p*<0.001 CTL-SC vs. DIAB-SC). Environmental enrichment increased the number of intersections in diabetic mice at the 20–140 µm region to levels similar to those found in control mice (*F*_housing(1,60)_ = 127.5, *p*<0.0001; *post hoc* test *p*<0.05 DIAB-SC vs. DIAB-EE; Fig. 5-B). A positive effect of the enriched environment was found at 160 µm and 180 µm from the soma in control animals (*F*_housing(1,60)_ = 39.5, *p*<0.0001; *post hoc* test *p*<0.01 and *p*<0.05 respectively CTL-SC vs. CTL-EE). Dendritic length was evaluated in three different concentric regions of the dendritic tree: 20 to 100 µm, 120 to 200 µm and 220 to 300 µm from the center of the soma (Fig. 5-C). This division rendered a good spatial resolution to identify changes in the dendritic trees. A statistically significant reduction of the dendritic length in the 20 to 100 µm region was found in diabetic mice exposed to standard caging (*F*_condition(1,16)_ = 3.852; *post hoc* test *p*<0.05 CTL-SC vs. DIAB-SC). Environmental enrichment prevented this reduction in diabetic mice (*F*_housing(1,16)_ = 8.621; *post hoc* test *p*<0.01 DIAB-SC vs. DIAB-EE). A main effect of ‘housing’ was found at the 120–200 µm and at the 220–300 µm ranges (*p*<0.01 and *p*<0.05 respectively).

### Dendritic spine density of CA1 pyramidal neurons

**Figure 6:**
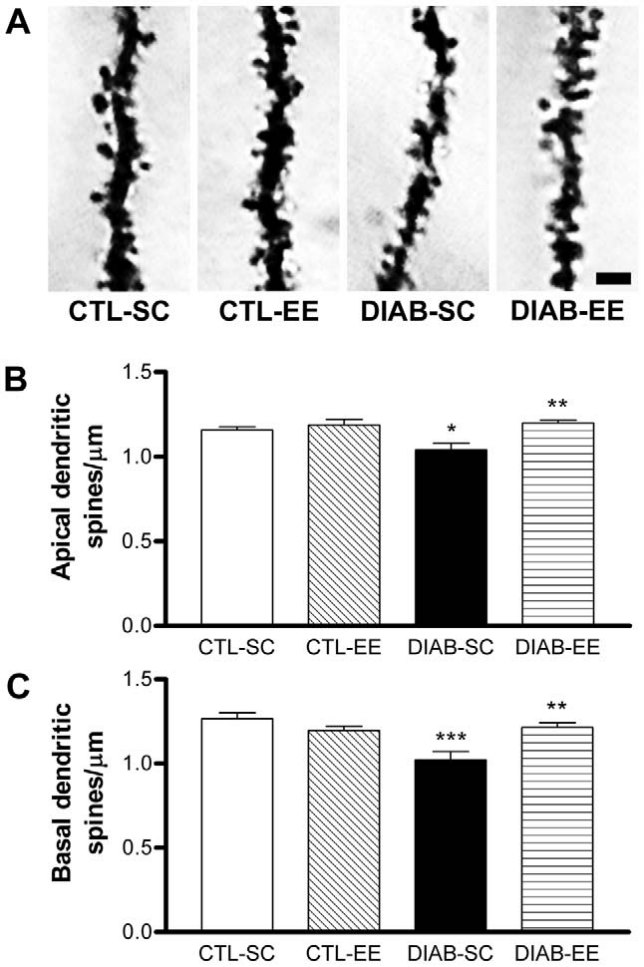
Dendritic spine density of CA1 pyramidal neurons. (A) Microphotographs of dendrites stained with the Golgi technique. Scale bar corresponds to 2 µm. (B) and (C) The quantification evidenced a reduction of the spine density in apical (B) and basal (C) dendrites in DIAB-SC mice (* *p*<0.05 and *** *p*<0.001 respectively vs. CTL-SC) with an increase in the DIAB-EE group (** *p*<0.01 for both apical and basal dendrites vs. DIAB-SC).

The spine density for both apical and basal dendritic trees of CA1 pyramidal cells was reduced in diabetic mice exposed to standard caging (Apical: *post hoc* test *p*<0.05; basal: *post hoc* test *p*<0.001; Fig. 6-B and 6-C respectively). Environmental enrichment was able to increase the spine density in diabetic mice (*post hoc* test *p*<0.01 for both apical and basal dendrites DIAB-SC vs. DIAB-EE) with no significant effect in control animals. A significant interaction was found between factors in both apical (*F*_interaction(1,16)_ = 4.784, *p*<0.05) and basal (*F*_interaction(1,16)_ = 13.96, *p*<0.01) dendrites.

### Vascular status of the dentate gyrus

**Figure 7:**
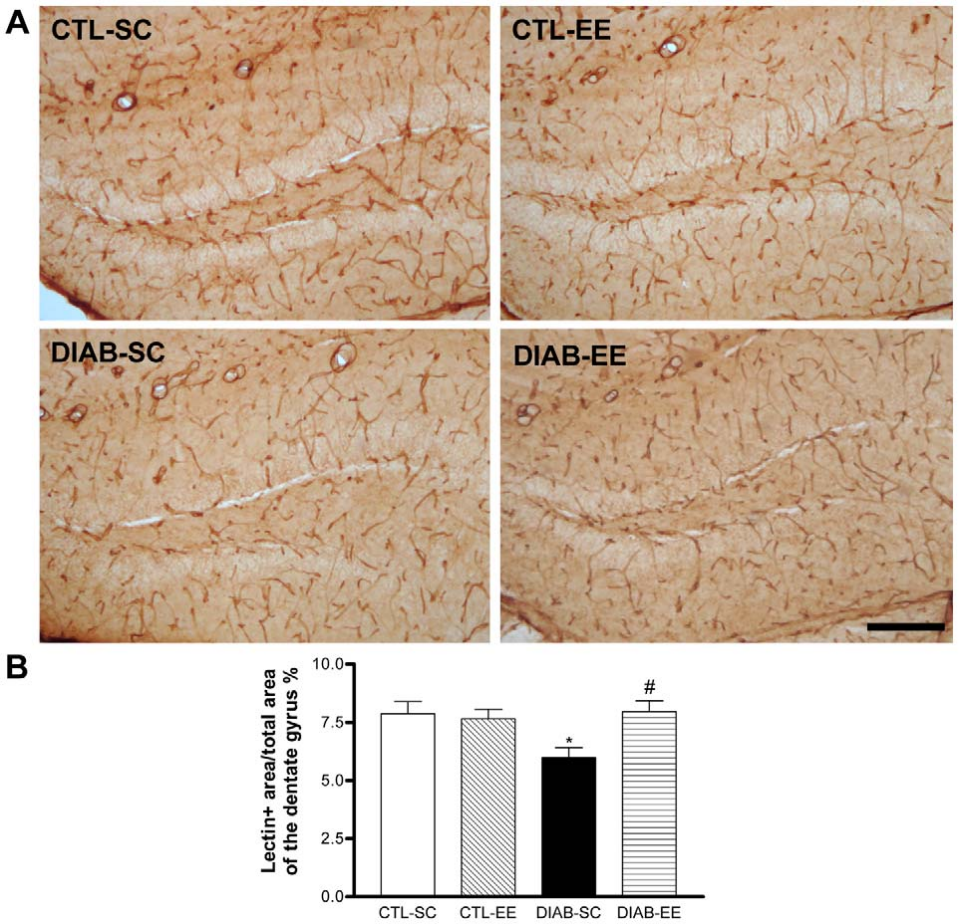
Vascular network of the dentate gyrus. (A) Representative microphotographs of the dentate gyrus showing the histochemistry for lectin Lycopersicon Esculentum. Scale bar corresponds to 100 µm. (B) Quantification of the lectin-positive fractional vascular area in the dentate gyrus. Results are expressed as the percentage of the area of the dentate gyrus occupied by blood vessels. Animals in the DIAB-SC group showed a smaller area percentage compared with CTL-SC mice (* *p*<0.05). Environmental enrichment positively regulated this parameter in diabetic mice (# *p*<0.05 vs. DIAB-SC).

Blood vessels were detected using lectin Lycopersicon Esculentum histochemistry on brain sections (Fig. 7). The positive area for this marker was measured in the dentate gyrus. The percentage of the dentate gyrus area covered by lectin-positive vessels was smaller in diabetic animals compared with controls (*post hoc* test *p*<0.05 CTL-SC vs. DIAB-SC) and the exposure of diabetic animals to an enriched environment increased it to control levels (*post hoc* test *p*<0.05 DIAB-SC vs. DIAB-EE; Fig. 7-B). An interaction was found between ‘condition’ and ‘housing’ factors (*F*_interaction(1,16)_ = 5.961, *p*<0.05). No differences were found in the dentate gyrus reference area between the 4 groups: CTL-SC = 363165±9807, CTL-EE = 365903±19905, DIAB-SC = 353145±18827, DIAB-EE = 379506±15170; data expressed in µm^2^).

## Discussion

The impact of the diabetic condition on the CNS, specifically on limbic structures, has been extensively studied by several groups including ours. These contributions suggested that hippocampal alterations, including impairments in adult neurogenesis, could be potentially associated with disturbances in cognitive functions and an increased risk of depression and dementia [1], [2], [19], [46]–[49].

On the other hand, environmental complexity induces multiple changes in the brain, promoting reorganization of sensorial cortices (i.e. visual, olfactory and tactile cortices develop in response to the exposure to environmental stimuli), enhancing synaptic plasticity (by increasing LTP), dendritic spine density and expression of synaptic proteins, receptors and neurotrophins [50]. The beneficial effects of environmental enrichment on the hippocampus have been described in animal models of physiological and pathological conditions [51], counting aging [52], stress [31], Alzheimer's disease [53], [54] and traumatic brain injury [55] among other situations, but, to our knowledge, this is the first report describing an environmental effect on the hippocampus in an animal model of diabetes.

Here, we present evidence of the positive effect that the exposure to a complex environment has on the hippocampus of diabetic mice. Cell proliferation, differentiation and survival of newborn neurons, vascularization of the dentate gyrus and dendritic complexity of mature hippocampal neurons were improved after a short exposure of streptozotocin-induced diabetic mice to an environment that incorporated social, sensorial and cognitive stimuli.

The evaluation of the cell proliferation, assessed by Ki67 immunodetection in the dentate gyrus, showed a tendency for diminishing in diabetic animals in standard caging and a significant increase with EE. A high proliferation rate has been associated with enrichment paradigms that specifically included physical exercise adding a wheel in the cage for voluntary running [50]. This component of the EE was purportedly excluded from our experiments to minimize effects of mixed stimuli. We found an increased proliferation after EE with limited physical exercise only in diabetic animals suggesting that environmental effects on this parameter become evident specifically under the pathological context. Survival of newly generated cells, studied by BrdU administration before starting the exposure to enriched or standard conditions, was markedly reduced in diabetic mice housed in normal cages, in agreement with previous results [9]. Surprisingly, a short period –ten days- of EE was able to increase cell survival in diabetic animals. Moreover, also as we have previously published, hippocampal volume is not affected by diabetes at 20 days of evolution [21].

Differentiation of newborn cells into a neuronal phenotype, evaluated as the percentage of BrdU-positive cells that showed TuJ1 co-staining, was diminished in diabetic mice. Potential mechanisms underlying this phenomenon could be an increased differentiation towards a glial phenotype, a delay in newborn cell differentiation and an accelerated maturation of TuJ1+ cells leading to the loss of the marker. A further thorough analysis of the cell differentiation dynamic should be done to elucidate this point. It is interesting to note that diabetic animals housed under enriched conditions showed neuronal differentiation levels similar to non-diabetic animals, demonstrating the capacity of sensorial and social stimuli to reverse disease-associated neuronal changes. The total number of granular neurons in the DG did not change between experimental groups Indicating that, at least in the studied temporal frame, diabetes or the differential housing conditions are not able to affect this factor.

Within the EE- induced changes in the dentate gyrus, our results showed an increase in the arborization of newly generated neurons. The dendritic length of DCX+ more mature neurons showed a decline in diabetic mice and a significant increase after exposure to the EE. Interestingly, cells expressing DCX, that will generate mature granule neurons, were described as specially responsive to certain enrichment paradigms [56], [57] and, in contrast, particularly vulnerable in conditions such as diabetes, aging and chronic stress [47], [58], [59].

Using a modified version of the Golgi silver impregnation technique we studied the dendritic ramification, dendritic length and spine density of hippocampal pyramidal neurons and these changes were prevented by EE. These parameters were clearly reduced in CA1 neurons of diabetic animals. Sholl analysis allowed us to conclude that the reduction in the dendritic ramification and length was more evident near the neuronal soma. There is probably a regional-dependent differential modulation of the dendritic tree by the diabetic condition. Brain derived neurotrophic factor (BDNF) is abundant in the hippocampus, among other cerebral structures, and its constitutive secretion is intimately involved with proximal processes growth [60]. Nitta et al have described a reduction in the brain content of BDNF protein and mRNA levels in experimental diabetes [48] and, in accordance, we found decreased BDNF mRNA levels in the hippocampus of streptozotocin-treated mice using *in situ* hybridization (unpublished results). Based on the data obtained in the present study, environmental enrichment seems to positively regulate the dendritic length of mature pyramidal neurons, being this effect more evident at zones that are proximal to the neuronal soma. Modulation of the dendritic complexity takes place at different levels and could be associated to changes in the synaptic function and also in animal behavior. Experimental diabetes is linked to cognitive impairment and behavioral changes together with hypothalamic-pituitary-adrenal axis dysregulation, including high plasma glucocorticoid levels, that could be coupled with the mentioned hippocampal disturbances [8], [46], [47], [49]. Along this line, a possible modulating effect of EE on the hypothalamic-pituitary-adrenal axis and related hormones in diabetic mice should not be discarded.

Another important finding of the present work is related to the brain vasculature in diabetic animals and the strong effect of EE. The lectin-positive area in the DG, specifically corresponding to blood vessel area, was lower in streptozotocin-treated mice housed in standard cages compared with controls. Concordantly, we have previously found a diminished vascular area in the granular cell layer of type 2 diabetic Goto-Kakisaki rats using von Willebrand factor immunohistochemistry [7]. This result constitutes a novel fact matching with other hippocampal disturbances associated with this disease. In this line, vessel abnormalities like vessel regression, hypoperfusion, endothelial degeneration, abnormal vessel growth, size and shape and microangiopathy are found in neurological disorders and are often associated with neuronal loss [61]. Neurogenesis in the adult dentate gyrus occurs in a neurovascular niche, where endothelial cells play a crucial role. Furthermore, a poor neurogenic potential has been linked to reduced vascular density in aging [62]. Improvement of cerebral blood flow could be a probable mechanism underlying the benefits of EE on the CNS, as the exposure to complex environments stimulates capillary branching and surface area [63] and promotes endothelial cell proliferation [64] in the brain. In addition, VEGF, a neurotrophic and pro-angiogenic factor, is upregulated after EE [65], possibly playing a role in the regulation of adult neurogenesis. A short term exposure -ten days- to an enriched environment was able to significantly increase the vessel area in the DG from diabetic mice. This last result, together with the effect on neuronal survival and dendritic development in new neurons, arborization and spine density in mature neurons constitute a clear evidence of the strong influence that experience exerts on hippocampal plasticity. Diabetic animals exposed to the enriched environment showed a slight but significant decrease in the glycemia. Though we can not discard a relationship of this effect with the multiple changes that environmental enrichment exerted in the hippocampus, it is important to consider that glycemia was over 28 mM in both diabetic groups, denoting a severe diabetic status. As reported by Stranahan *et al* [46] in streptozotocin-induced diabetic rats, increased glucose circulating levels do not correlate with changes in glucose or insulin levels in the hippocampus, indicating an indirect mechanism of damage. Possible intermediate pathways could be an increased activity of the HPA axis [46], [49], upregulation of the receptor for advanced glycation end products (RAGE) expression in the brain [66], brain vascular inflammation [67] and increased oxidative stress [68]. In a previous study done in spontaneously type 1 diabetic mice (NOD mice), we found that hippocampal abnormalities are evident before the onset of hyperglycemia, indicating that other pathological, glycemia-independent pathways are activated [9].

Notably, in our experiments, control animals exposed to EE did not show significant differences in the studied parameters compared to standard-housed mice, with the exception of an increase in the dendritic length of DCX+ cells. Doublecortin+ cells in the dentate gyrus seem to be particularly sensitive to changes in the environment even in normal animals. Possibly, a prolonged exposure time may be required for changes to become evident in normal, young animals.

Remarkably, in the literature there is no complete consensus about the protocols of EE used by different research groups. Differences can be found regarding objects included, duration of EE, cage size, type of enrichment and the combination or not of EE with physical exercise [50]. Running can promote neurogenesis, synaptic plasticity and learning [69]. In streptozotocin-induced diabetic rats, treadmill running was shown to improve synaptic plasticity in the DG [70]. However, physical exercise alone may not be able to promote morphological changes in the dendritic trees of hippocampal neurons [32]. Several similarities in the effects of both aging and diabetes can be found in the hippocampus [58], [71] and an interaction between diabetes and aging was described [3]. As in aging, increased oxidative stress is present in the diabetic brain [68] and constitutes another factor leading to the diabetes-induced behavioral deficits [72], [73]. In aged rats, EE was shown to potentially protect against oxidative stress and to improve cognitive performance [74]. As mentioned, the duration of EE is a key factor to be considered when brain plasticity is studied. Bindu and colleagues demonstrated that a short-term exposure to an EE was able to enhance dendritic branching in ventral subicular-lesioned rats while few changes were found on control animals [30]. In agreement to that, our results suggest that in a pathological situation with several hippocampal disturbances, like type 1 diabetes, a short term exposure to EE could be enough to promote a potent brain response involving an enhancement of adult neurogenesis, dendritic growth and spine density as well as an increase of the hippocampal vascular area.

Our data could represent a new approach to the prevention of some of the central nervous system complications correlating with type 1 diabetes. Even though learning and memory testing is still needed to link these results with behavioral changes in streptozotocin-treated mice, the diabetic brain reveals that it is still able to react in response to environmental challenges.
